# Bayesian inference for integrated pharmacokinetic modelling of mitragynine and 7-hydroxymitragynine

**DOI:** 10.5599/admet.3170

**Published:** 2026-03-06

**Authors:** Dion Notario, Untung Gunawan, Pretty Falena Atmanda Kambira, Erna Wulandari, Eko Adi Prasetyanto

**Affiliations:** 1 Department of Pharmacy, School of Medicine and Health Sciences, Atma Jaya Catholic University of Indonesia, Jalan Pluit Utara no 2, Jakarta Utara, 14440, Indonesia; 2 Research Center for Cheminformatics and Molecular Modeling, Atma Jaya Catholic University of Indonesia, Jakarta, 14440, Indonesia; 3 The Indonesian Center for Drug Research (ICDR), Atma Jaya Catholic University of Indonesia, Jakarta, 14440, Indonesia

**Keywords:** Kratom alkaloids, linked parent-metabolite model, Markov Chain Monte Carlo, multi-compartment disposition, steady-state simulation

## Abstract

**Background and purpose:**

Mitragynine is an active compound in kratom that is metabolized to the pharmacologically active 7-hydroxymitragynine, requiring an integrated pharmacokinetic approach to maintain plasma concentrations of both within the optimal range. This study aims to develop an integrated pharmacokinetic model of mitragynine and 7-hydroxymitragynine using Bayesian inference.

**Experimental approach:**

A secondary dataset of mitragynine and 7-hydroxymitragynine in healthy human plasma was extracted and used to construct a two-compartment pharmacokinetic model upon oral administration. Initial parameter estimation was performed using a deterministic model fit to determine prior parameters. Bayesian inference was performed using Hamiltonian Monte Carlo across four independent chains, each with 2,000 iterations.

**Key results:**

The prior distribution estimation indicated that the Markov Chain Monte Carlo chain had converged and attained stationarity, yielding many independent effective samples. In general, no correlation between pharmacokinetic parameters was found due to modelling errors. The posterior predictive check plot confirmed a good fit between the model and the data. Pharmacokinetic simulations of repeated administration have been successfully developed and used to predict essential parameters in repeated administration, such as accumulation factors, maximum plasma concentration, time to maximum concentration, minimum plasma concentration, and area under the curve.

**Conclusion:**

The pharmacokinetics of mitragynine and 7-hydroxymitragynine were successfully modelled simultaneously with two compartments and proportional residuals using Bayesian inference with high accuracy.

## Introduction

Kratom (*Mitragyna speciosa*) is a psychoactive plant widely consumed in the United States, with a prevalence of approximately 9.1 % [1]. Kratom is widely used for various reasons, such as improving quality of life, boosting energy, replacing opioids or alcohol, and alleviating opioid withdrawal symptoms [2]. However, improper use can cause undesirable effects such as addiction, hepatitis, and withdrawal symptoms [3-5]. This makes the use of kratom leaves in humans still controversial [6].

Mitragynine is the bioactive compound responsible for the pharmacological effects of kratom leaves [7]. Mitragynine is metabolized in the liver by CYP3A4 to the active metabolite 7-hydroxymitragynine [8]. To determine a safe and effective dose of kratom, pharmacokinetic-pharmacodynamic modeling of mitragynine and 7-hydroxymitragynine in humans is crucial [9]. However, the pharmacokinetics of mitragynine and 7-hydroxymitragynine have been modeled separately as reported by Heutis *et al.* [10], Trakulsrichai *et al*. [11] and Methaneethorn *et al*. [12].

Therefore, this study aims to develop a single integrated pharmacokinetic model for mitragynine and 7-hydroxymitragynine. We simultaneously modeled the pharmacokinetics of mitragynine and 7-hydroxymitragynine using a single set of differential equations, thereby providing a more mechanistic model and integrated interpretation. Parameter estimation was performed using Bayesian inference to generate robust parameters, even when using weakly informative priors [13]. The pharmacokinetic model and posterior parameter estimates from this study will serve as a basis for designing clinical trials in larger populations to generate safer, more effective model-informed dose selections.

## Experimental

### Data acquisition

Pharmacokinetic data, including the time, h; mean concentration, ng·mL^-1^; and standard deviation of mean concentration, were obtained from Mongar *et al.* licensed under CC BY-NC-ND 4.0. [14,15]. A dataset from a pharmacokinetic plot was extracted using WebPlotDigitizer and saved as an .xlsx file. The 7-hydroxymitragynine data were excluded at hour 35 because it was identified as an outlier by visual inspection.

### Pharmacokinetic model

**Figure 1.**
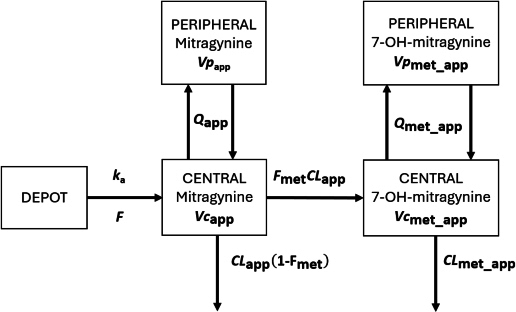
Pharmacokinetic model of mitragynine and 7-hydroxymitragynine



The pharmacokinetic models for mitragynine and 7-hydroxymitragynine were built using a linked parent-metabolite framework with central and peripheral compartments, as shown in Figure 1 [16]. Mitragynine and its active metabolite, 7-hydroxymitragynine, were modelled using the following system of differential Equations (1) to (5):

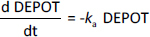

(1)




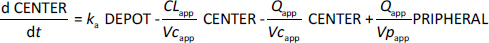

(2)




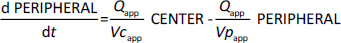

(3)




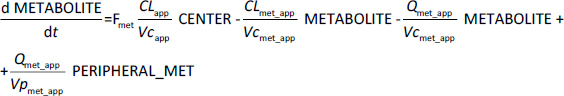

(4)




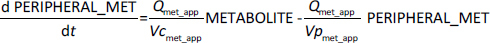

(5)


where *t* / h is time after first dose, *k*_a_ / h^-1^ is first order absorption constant, DEPOT, mg is amount of mitragynine in absorption site, CENTER, mg is amount of mitragynine in central compartment (plasma and highly perfused tissues), *CL*_app_ / L·h^-1^ is apparent total clearance of mitragynine, *Vc*_app_ / L is apparent volume distribution of mitragynine for the central compartment, PERIPHERAL, mg is amount of mitragynine in peripheral compartment (poorly perfused tissues), *Q*_app_ / L·h^-1^ is apparent intercompartment clearance of mitragynine, *Vp*_app_ / L is apparent volume distribution of mitragynine for the peripheral compartment, METABOLITE / mg is amount of 7-hydroxymitragynine in central compartment, *F*_met_ is fraction of mitragynine in the central compartment converted to 7-hydroxymitragynine, *CL*_met_app_ / L·h^-1^ is apparent total clearance of 7-hydroxymitragynine, *Vc*_met_app_ / L is apparent volume distribution of 7-hydroxymitragynine in central compartment, *Q*_met_app_ / L·h^-1^ is apparent intercompartment clearance of 7-hydroxymitragynine, PERIPHERAL_MET, mg is amount of 7-hydroxymitragynine in peripheral tissue, *Vp*_met_app_ / L is apparent volume distribution of 7-hydroxymitragynine for the peripheral compartment.

### Initial parameter estimation

All statistical operations, model fitting, and visualizations were performed using R 4.4.2 and accessed through the RStudio 2024.12.0.467 [17,18]. Prior to Bayesian inference, initial parameter estimates were obtained through deterministic model fitting using the rxode2 and FME packages [19,20].

Model simulation was performed using rxSolve() command in rxode2 [19], and visual comparisons between simulated and observed data were generated using ggplot2 [21]. Simultaneous fitting of mitragynine and 7-hydroxymitragine data was conducted using a custom cost function that minimized the weighted sum of squared errors on the log-transformed concentration scale.

The vector of pharmacokinetic parameters is denoted by **θ**. The cost function, Cost(**θ**), is defined as the weighted sum of squared residual errors (SSE) for mitragynine and 7-hydroxymitragynine, based on the log-transformed plasma concentrations. We applied a penalty rule whereby the cost function is assigned a value of 10^10^ whenever the calculation of Cost(**θ**) results in an undefined or infinite value. The cost function is mathematically expressed in Equation (6).



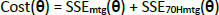

(6)


SSE_mtg_ and SSE_7Ohmtg_ denote the squared error sums of the log-transformed mean plasma concentrations of mitragynine and 7-hydroxymitragynine, respectively, derived from Equation (7).



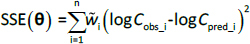

(7)


where *C*_obs_i_ and *C*_pred_i_ are the mean observed and predicted plasma concentrations, respectively. The log function in Equation (7) is the natural logarithm, in accordance with the implementation in R.

Each observation's coefficient of variation was used to derive weights (*w̃*_i_), which were normalized to guarantee a balanced contribution across all time points. The coefficient of variations (CV_i_) was calculated based on the standard deviation (*σ*_obs_i_) and mean observed plasma concentration according to Equation (8) with a minimum limit of 0.05.



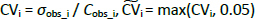

(8)


The weighting factors were calculated as the inverse of CVi and then normalized by the mean weighting factor, as shown in Equations (9) and (10).



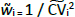

(9)




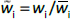

(10)


We used the optimize() function with the L-BFGS-B algorithm to optimize the parameters [22], making sure the values were not biologically impossible by setting realistic limits for each. The ultimate parameter estimates derived from this deterministic fitting provided the foundation for developing prior distributions in the following Bayesian analysis, except for the proportional residual error prior, which is defined as a weakly informative normal distribution [23].

### Bayesian parameter estimation

Pharmacokinetic parameters were estimated using a Bayesian framework implemented in Stan via the cmdstanr package [24]. The ODE system was solved using the Runge-Kutta 4(5) method (ode_rk45) for both parent and metabolite concentration timepoints [25]. Predicted plasma concentrations (*C*_pred_i_) for both the parent compound and its metabolite were derived by numerically solving a system of ordinary differential equations (ODEs) representing a two-compartment model for each entity. Predicted concentrations were scaled to ng·mL^-1^ based on the respective central volumes of distribution.

We implemented a residual error model that combines the standard deviation of the observed mean concentrations (*σ*_obs_) with a proportional residual error (*σ*_prop_) to account for inter-individual variability. The combined residual error was defined by Equation (11) as:



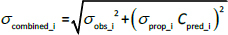

(11)


Observed concentrations (*y*_i_) were assumed to follow a log-normal distribution, Equation (12):



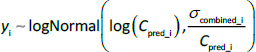

(13)


### Posterior sampling and diagnostics

Posterior distributions were estimated using Hamiltonian Monte Carlo (HMC) sampling, implemented across four independent chains, each with 2,000 iterations to ensure convergence. Model diagnostics, including the potential scale reduction factor (*R̂*), trace plots, and effective sample size (ESS), were assessed to confirm the reliability of the sampling process. Posterior predictive checks were conducted using replicated datasets drawn from the posterior distributions to evaluate the model's predictive performance.

### Pharmacokinetic simulation

The pharmacokinetic profiles of mitragynine and 7-hydroxymitragynine were simulated using the rxSolve() function. The simulations were based on a previously developed multi-compartment model. All parameter values were taken from prior Bayesian fitting to observed pharmacokinetic data, ensuring that the simulations reflect realistic variability.

Posterior distributions were obtained by updating prior distributions with single-dose pharmacokinetic data during model fitting. We randomly sampled 200 parameter sets from the posterior distributions obtained during model fitting to capture uncertainty. These samples were then used to simulate repeated oral dosing of mitragynine at 25 mg every 24 h for seven doses. Concentration *vs* time profiles were generated over a 200 h period with 0.5 h intervals.

The median predicted concentration and the 95 % credible interval (2.5^th^ to 97.5^th^ percentiles) were calculated for both mitragynine and 7-hydroxymitragynine for each time point. Results were visualized using ggplot2, with profiles shown on both linear and logarithmic scales. Shaded ribbons represent uncertainty bounds, highlighting variability across the posterior simulations. Key parameters for each administered dose, such as maximum plasma concentration (*C*_max_), minimum plasma concentration (*C*_min_), and time to maximum concentration (*t*_max_), were extracted from the simulation data, while the area under the curve (AUC) was calculated using the trapezoidal rule. The accumulation factor is calculated by dividing the *C*_max_ at the last dose at steady state by the first dose.

## Results and discussion

The results of prior parameter estimation are presented in Table 1, except for the priors *σ*_prop_parent_ and *σ*_prop_met_, which are set as weakly informative normal distributions [23]. Based on these prior assumptions, we have successfully estimated the pharmacokinetic parameters for mitragynine and 7-hydroxymitragynine, summarized in Table 2. The Gelman-Rubin statistic (*R̂*) is close to 1, indicating that the Markov Chain Monte Carlo (MCMC) chain has converged. The ESS values for all parameters are above 1,000, indicating that MCMC sampling has produced many independent effective samples.

**Table 1.** Prior assumption

Parameter	Mean	Standard deviation	Distribution
ln (*k*_a_ / h^-1^)	0.60	0.41	log-normal
ln (*CL*_app_ / L·h^-1^)	3.00	0.33	log-normal
ln (*Vc*_app_ / L)	4.99	0.39	log-normal
ln (*Q*_app_ / L·h^-1^)	2.39	0.42	log-normal
ln (*Vp*_app_ / L)	5.77	0.36	log-normal
ln (*CL*_met_app_ / L·h^-1^)	4.58	0.33	log-normal
ln (*Vc*_met_app_ / L)	4.40	0.56	log-normal
ln (*Q*_met_app_ / L·h^-1^)	2.09	0.77	log-normal
ln (*Vp*_met_app_ / L)	4.41	0.78	log-normal
ln *F*_met_	-0.48	0.36	log-normal
*σprop_parent	0	0.5	normal
*σprop_met	0	0.5	normal

*The prior of *σ*_prop_parent_ and *σ*_prop_met_ was defined as a weakly informative normal distribution, *σ*_prop_ ~N(0,5).

**Table 2.** Estimated pharmacokinetic parameters

Parameters	Mean	Median	Standard deviation	95 % credible interval		*R̂*	ESS
				Lower	Upper		
*k*_a_ / h^-1^	2.11	1.99	0.72	1.05	3.92	1.00	3242
*CL*_app_ / L·h^-1^	18.01	17.85	2.23	14.08	22.86	1.00	5820
*Vc*_app_ / L·h^-1^	117.98	115.71	21.26	82.08	165.48	1.00	4782
*Q*_app_ / L·h^-1^	11.03	10.76	2.08	7.74	15.94	1.00	4092
*Vp*_app_ / L	358.87	355.64	69.81	230.25	487.40	1.00	3055
CL_met_app_ / L·h^-1^	108.03	106.29	23.47	67.38	156.60	1.00	3469
*Vc*_met_app_ / L	57.37	50.61	28.49	23.01	130.45	1.00	3231
*Q*_met_app_ / L·h^-1^	10.11	8.52	6.54	1.83	26.53	1.00	2955
*Vp*_met_app_ / L	87.57	71.76	58.62	17.82	248.95	1.00	3797
*F*_met_	0.66	0.65	0.14	0.41	0.95	1.00	2858
*σ*_prop_parent_	0.17	0.14	0.13	0.01	0.49	1.00	3020
*σ*_prop_met_	0.13	0.11	0.10	0.01	0.39	1.00	3406

**Figure 2.**
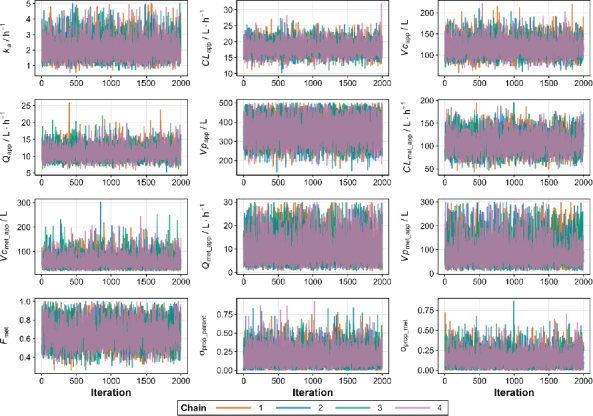
Trace plots of Markov Chain Monte Carlo (MCMC) samples for pharmacokinetic parameters

**Figure 3.**
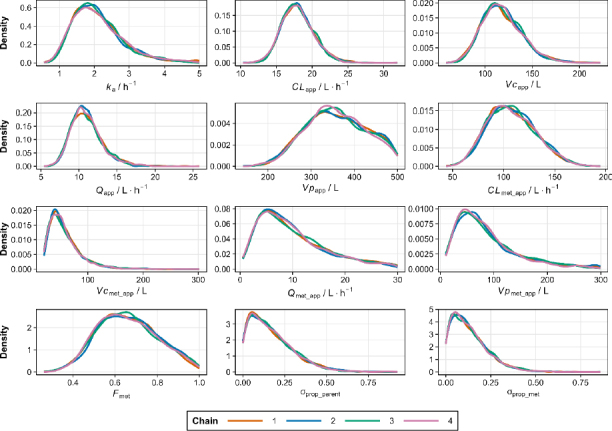
Overlay posterior density plot for pharmacokinetic parameters

**Figure 4.**
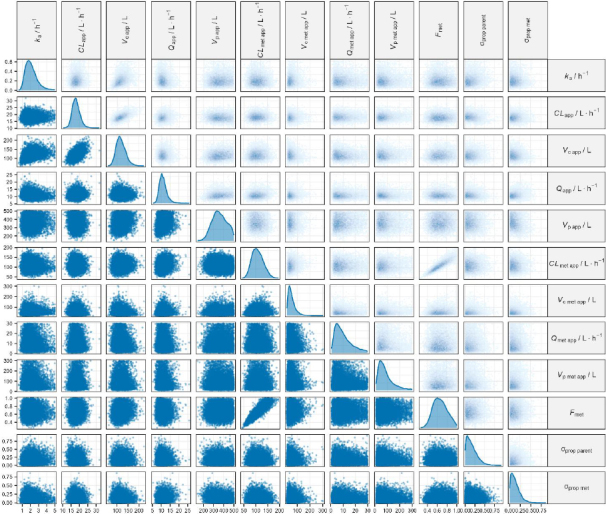
Pairwise scatter plot of posterior samples

**Figure 5.**
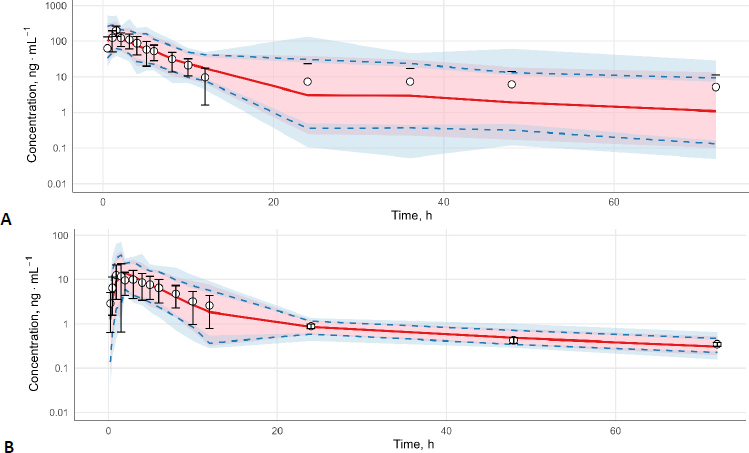
Posterior predictive check of mitragynine (A) and 7-hydroxymitragynine (B). Shaded light pink areas represent the 50 % prediction interval, spanning from the 25^th^ to the 75^th^ percentiles and solid red lines indicate the median predictions (50^th^ percentiles). The blue area shows the 90 % prediction interval, which spans between the 5^th^ to 95^th^ percentiles and the blue dashed lines indicate the 5^th^ and 95^th^ percentiles. Observed plasma concentrations (open circles with error bars) are overlaid for comparison

These results (Table 2) were validated by the profile trace plot and overlayed posterior density plots for stable MCMC chains (Figures 2 and 3). Each chain has mixed, and there is no clear upward or downward trend, suggesting that sampling has become stationary. Pairwise scatter plots of posterior samples show that, in general, there is no correlation between parameters, except between *CL*_app_ with *Vc*_app_ and *F*_met_ with *CL*_met_app_ (Figure 4). However, this correlation arises from the mathematical relationship in the pharmacokinetic model, not from modelling errors [26,27]. The posterior predictive check plot (Figure 5) shows that the observed data points fall within the 2.5^th^ to 97.5^th^ percentile range. This indicates that the model is well calibrated to the data, has no potential for serious misfit, and captures reasonable variability and uncertainty well.

The estimated median *CL*_app_ and *C*_max_ of mitragynine following a single dose were 17.85 (95 % credible interval: 14.08 to 22.86) L·h^-1^ and 158 (95 % credible interval: 95 to 279) ng·mL^-1^, which are comparable to 24.90 (95 % confidence interval: 16.17 to 52.65) L·h^-1^ and 159.12 (95 % confidence interval:134.06 to 217.44) ng·mL^-1^ reported in previous non-compartmental analyses at comparable doses [14]. The *C*_max_ level of 7-hydroxymitragynine after a single dose in this study was 15 (min to max: 10 to 21) ng·mL^-1^, almost identical to the 12.81 (95 % confidence interval: 9.47 to 22.23) ng·mL^-1^ in a prior study [14].

Apparent volume distribution of mitragynine (*Vc*_app_) and 7-hydroxymitragynine (*Vp*_met_app_) estimated from our study was 115.71 (95 % credible interval: 82.08 to 165.48) L and 50.61 (95% credible interval: 23.01 to 130.45) L, respectively, which is substantially different from the values reported at 521.1 (95 % confidence interval: 394.7-858.1) and 23.01 (95 % confidence interval: -885.11 to 2518.79) L [14]. The estimated apparent total body clearance (*CL*_met_app_) of 7-hydroxymitragynine was 106.29 (95 % credible interval: 67.38 to 156.60), which is considerably higher than 0.84 (95 % confidence interval: -5.61 to 19.07) in the previous study [14]. This discrepancy was expected because non-compartmental analysis assumes a linear model with no metabolic conversion or tissue partitioning. Moreover, the negative values in the 95 % confidence intervals for the geometric mean *Vp*_met_app_ and *CL*_met_app_ using the non-compartmental method indicate methodological issues in the parameter estimation process. In contrast, our approach accounted for tissue partitioning and metabolic conversion and quantified uncertainty using posterior distributions, yielding more mechanistic results and relevant interpretations of mitragynine disposition.

**Figure 6.**
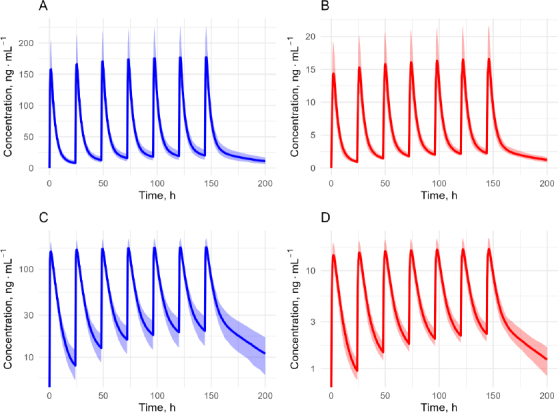
Simulated plasma concentration vs time profiles of mitragynine and 7-hydroxymitragynine following repeated oral administration of mitragynine (25 mg every 24 hours). (A) Mitragynine on a linear scale; (B) 7-hydroxymitragynine on a linear scale; (C) Mitragynine on a semi-logarithmic scale; (D) 7-hydroxymitragynine on a semi-logarithmic scale. Shaded regions represent the 95 % prediction interval of the predicted concentrations

Pharmacokinetic simulations based on the pharmacokinetic parameters obtained from the posterior distribution were successfully performed (Figure 6). Visual inspection of the pharmacokinetic profiles revealed median plasma accumulation factors of mitragynine and 7-hydroxymitragynine of 1.13 (min = 1.08, max = 1.31) and 1.14 (min = 1.09, max = 1.35), respectively. Accumulation factor values close to one indicate low plasma accumulation of mitragynine and 7-hydroxymitragynine. Peak time (*t*_max_) was calculated from the time of the previous dose, not the initial dose. We found that median values of *t*_max_ of mitragynine and 7-hydroxymitragynine were 1.5 (min = 1, max = 2) hours and 2 (min = 1.5, max = 3.5) hours, respectively. Key parameters such as *C*_max_, *C*_min_, and AUC were predictable, and the results are summarized in Tables 3 and 4.

**Table 3.** Prediction *C*_max_, *C*_min_ and AUC of mitragynine

Dosing event	*C*_max_ / ng·mL^-1^			*C*_min_ / ng·mL^-1^			AUC, ng·h·mL^-1^		
	Median	Minimal	Maximal	Median	Minimal	Maximal	Median	Minimal	Maximal
1 (0 h)	157.96	95.24	279.20	0.00	0.00	0.00	983.47	711.23	1349.42
2 (24 h)	165.75	101.51	290.69	7.66	4.30	15.25	1118.57	839.52	1573.94
3 (48 h)	170.77	104.65	298.42	12.32	6.81	24.14	1204.64	904.27	1726.56
4 (72 h)	173.93	106.74	303.69	15.28	8.60	29.38	1260.31	937.14	1830.58
5 (96 h)	175.73	108.15	307.28	17.19	9.88	32.47	1296.46	953.83	1901.46
6 (120 h)	176.75	109.09	309.72	18.55	10.79	34.29	1324.66	962.30	1949.77
7 (144 h)	177.66	109.73	311.39	19.53	11.44	35.36	1343.60	966.60	1982.69

Mitragynine is administered orally at a dose of 25 mg every 24 hours

**Table 4.** Prediction *C*_max_, *C*_min_, and AUC of 7-hydroxymitragynine

Dosing event	*C*_max_ / ng·mL^-1^			*C*_min_ / ng·mL^-1^			AUC, ng·h·mL^-1^		
	Median	Min	Max	Median	Min	Max	Median	Min	Max
1 (0 h)	14.70	9.76	20.59	0.00	0.00	0.00	106.14	74.81	136.40
2 (24 h)	15.58	10.54	21.95	0.94	0.70	1.73	122.04	92.18	162.66
3 (48 h)	16.09	11.06	22.77	1.48	1.15	2.65	132.45	101.25	177.44
4 (72 h)	16.41	11.43	23.18	1.83	1.45	3.20	139.27	107.54	184.95
5 (96 h)	16.64	11.70	23.39	2.06	1.63	3.52	143.79	111.06	188.82
6 (120 h)	16.78	11.89	23.50	2.19	1.74	3.71	146.93	112.99	190.85
7 (144 h)	16.88	11.99	23.56	2.30	1.81	3.82	148.92	114.14	191.94

Mitragynine is administered orally at a dose of 25 mg every 24 hours

There are no regulations from any country or global organization that set explicit thresholds for the plasma levels of mitragynine and 7-hydroxymitragynine. However, some researchers have reported that mitragynine and 7-hydroxymitragynine concentrations of >1,000 and 150 ng·mL^-1^, respectively, are associated with death from kratom overdose [28,29]. In another study, it was reported that mitragynine and 7-hydroxymitragynine did not cause abnormal symptoms in all volunteers with *C*_max_ the highest 125 and 22.7 ng·mL^-1^, respectively [10]. Based on these data and the pharmacokinetic models of mitragynine and 7-hydroxymitragynine, more precise dosage settings can be designed to achieve safe and effective concentrations by adjusting doses and administration intervals in the simulation parameters.

This study has some limitations that should be considered when interpreting the findings. This study was based on secondary data, with limited access to detailed clinical data, such as mitragynine and 7-hydroxymitragynine concentrations for each individual. This leaves the effects of covariates on pharmacokinetic parameters unknown, which may impact the generalizability of these parameters. Further studies with primary data are needed to estimate the effects of covariates in the population.

## Conclusions

The pharmacokinetics of mitragynine and its active metabolite, 7-hydroxymitragynine, were successfully modeled simultaneously with two compartments and proportional residuals using Bayesian inference with high accuracy. This approach provides pharmacokinetic parameters and credible intervals that can be used to simulate realistic pharmacokinetic profiles. This model was built and validated using secondary data, with limited access to individual-level concentration data, preventing the calculation of covariate effects. Further research with individual concentration-time data is needed to estimate the effects of covariates and build more robust simulations that can be generalized to the population, resulting in safe and effective model-based kratom dosage adjustments.
